# Author Correction: Genomic recombination events may reveal the evolution of coronavirus and the origin of SARS-CoV-2

**DOI:** 10.1038/s41598-021-02549-9

**Published:** 2021-11-23

**Authors:** Zhenglin Zhu, Kaiwen Meng, Geng Meng

**Affiliations:** 1 School of Life Sciences, Chongqing University, No.55 Daxuecheng South Road, Shapingba, Chongqing, 401331 China; 2 College of Veterinary Medicine, China Agricultural University, Beijing, 100094 China

Correction to: *Scientific Reports* 10.1038/s41598-020-78703-6, published online 10 December 2020

The original version of this Article contained errors.

During revision of some of the nomenclature in the manuscript the Authors introduced errors in the description of recombination events, suggesting that SARS-CoV-2 is a DNA virus. SARS-CoV-2 is an RNA virus; the Article has been corrected as follows.

In the Abstract,

"Population genetic analyses provide estimates suggesting that the putative introduced DNA within the RBD is undergoing directional evolution."

now reads:

"Population genetic analyses provide estimates suggesting that the putative introduced genetic sequence within the RBD is undergoing directional evolution."

In the Results, subheading 'Recombination between bat and pangolin coronaviruses may represent to the origin of SARS-CoV-2',

"One of these two recombinationally intergrated DNA fragments is located inside polyprotein 1ab (pp1ab, open reading frame 1 (ORF1)), referred to as RI_DNA_ORF1 in this manuscript, and the other fragment spans the 3′ end of ORF1 and the 5′ beginning of the S protein, referred to as RI_DNA_Boundary in this manuscript (Fig. 2A)."

now reads:

"One of these two recombinationally intergrated RNA fragments is located inside polyprotein 1ab (pp1ab, open reading frame 1 (ORF1)), referred to as RI_RNA_ORF1 in this manuscript, and the other fragment spans the 3’ end of ORF1 and the 5’ beginning of the S protein, referred to as RI_RNA_Boundary in this manuscript (Figure 2A)."

and

"Our results suggested with high probability that SARS-CoV-2 originated from a bat coronavirus after recombinational integration of a DNA fragment from a pangolin coronavirus into the S protein gene (Fig. 2B). This putative integrated DNA fragment, referred to as RI_DNA_S in this manuscript, encodes a 76 AA long peptide and is located in the RBD (Fig. S2), which may influence the host preference of the virus."

now reads:

"Our results suggested with high probability that SARS-CoV-2 originated from a bat coronavirus after recombinational integration of a RNA fragment from a pangolin coronavirus into the S protein gene (Figure 2B). This putative integrated RNA fragment, referred to as RI_RNA_S in this manuscript, encodes a 76 AA long peptide and is located in the RBD (Figure S2), which may influence the host preference of the virus."

In Figure 2 legend,

"Coordinate positions or positions of three recombinationally integrated DNA regions (indicated out by orange dotted lines) in the genome of SARS-CoV-2 (MN908947), with major proteins marked. ‘a’, ’b’ and ‘c’ refer to RI_DNA_ORF1, RI_DNA_Boundary and RI_DNA_S, respectively."

now reads:

"Coordinate positions or positions of three recombinationally integrated RNA regions (indicated out by orange dotted lines) in the genome of SARS-CoV-2 (MN908947), with major proteins marked. ‘a’, ’b’ and ‘c’ refer to RI_RNA_ORF1, RI_RNA_Boundary and RI_RNA_S, respectively."

Additionally, throughout the Results, the Discussion, the Methods sections, in Figures 1 and 2, all figure legends, in Table 2 and the table legend, and in the Supplementary Files all instances of RI_DNA_ORF1, RI_DNA_Boundary, and RI_DNA_S have been replaced with RI_RNA_ORF1, RI_RNA_Boundary, and RI_RNA_S, respectively.

Finally, since RDP v4 was used in this study, Reference 32, which was:

Martin, D. P. et al. RDP3: a flexible and fast computer program for analyzing recombination. Bioinformatics 26, 2462–2463 (2010).

now reads:

Martin, D. P. et al. RDP4: Detection and analysis of recombination patterns in virus genomes. Virus Evol 1, vev003 (2015).

The errors have been corrected in the original Article and in the Supplementary Information file that accompanies the original Article.

